# Engineering an Enhanced, Thermostable, Monomeric Bacterial Luciferase Gene As a Reporter in Plant Protoplasts

**DOI:** 10.1371/journal.pone.0107885

**Published:** 2014-10-01

**Authors:** Boyu Cui, Lifeng Zhang, Yunhong Song, Jinsong Wei, Changfu Li, Tietao Wang, Yao Wang, Tianyong Zhao, Xihui Shen

**Affiliations:** 1 State Key Laboratory of Crop Stress Biology for Arid Areas and College of Life Sciences, Northwest A&F University, Yangling, Shaanxi, China; 2 Department of Microbiology, College of Life Sciences, Northwest A&F University, Yangling, Shaanxi, China; 3 Department of Biochemistry and Molecular Biology, College of Life Sciences, Northwest A&F University, Yangling, Shaanxi, China; Universidad Miguel Hernández de Elche, Spain

## Abstract

The application of the *luxCDABE* operon of the bioluminescent bacterium *Photorhabdus luminescens* as a reporter has been published for bacteria, yeast and mammalian cells. We report here the optimization of fused *luxAB* (the bacterial luciferase heterodimeric enzyme) expression, quantum yield and its application as a reporter gene in plant protoplasts. The fused *luxAB* gene was mutated by error prone PCR or chemical mutagenesis and screened for enhanced luciferase activity utilizing decanal as substrate. Positive *luxAB* mutants with superior quantum yield were subsequently shuffled by DNase I digestion and PCR assembly for generation of recombinants with additional increases in luciferase activity in bacteria. The coding sequence of the best recombinant, called e*luxAB*, was then optimized further to conform to *Arabidopsis* (*Arabidopsis thaliana*) codon usage. A plant expression vector of the final, optimized e*luxAB* gene (opt-e*luxAB*) was constructed and transformed into protoplasts of *Arabidopsis* and maize (*Zea mays*). Luciferase activity was dramatically increased for opt-e*luxAB* compared to the original *luxAB* in *Arabidopsis* and maize cells. The opt-e*luxAB* driven by two copies of the 35S promoter expresses significantly higher than that driven by a single copy. These results indicate that the e*luxAB* gene can be used as a reporter in plant protoplasts. To our knowledge, this is the first report to engineer the bacterium *Photorhabdus luminescens* luciferase *luxAB* as a reporter by directed evolution which paved the way for further improving the *luxAB* reporter in the future.

## Introduction

Reporter genes are valuable tools for promoter analysis [1], imaging of gene expression [2], [3], detecting xenobiotic compounds [4], protein subcellular localization [5], protein-protein interactions [6], and discovery of genes as potential targets for disease [7]. Although the current reporter gene assay systems, such as GFP, firefly luciferase (Fluc), LacZ, CAT and GUS have greatly advanced molecular biology research, all except the bacterial luciferase system require additional manipulations such as cell lysis, substrate addition or UV excitation. A distinct advantage of the bacterial luciferase (*lux*) system is the ability to utilize substrates readily available in the cell (FMNH_2_, O_2_, and a fatty aldehyde), thus eliminating the need for cell lysis or exogenous substrate addition [8], [9]. Alternatively, the substrate decanal can be exogenously provided without disruption of the cell, as decanal traverses cell membranes easily. Therefore, using a bacterial luciferase-based reporter allows investigators to non-invasively study gene expression or physiological changes in viable cells in real-time. In addition, the use of a bacterial luciferase-based reporter is more convenient and economical, especially for large-scale experiments. For these reasons, development of a reliable, bacterial luciferase-based reporter for eukaryotic cells would be extremely useful especially for continuous *in vivo* real-time assays.

Many attempts have been made for expression of the bacterial *lux* system in different eukaryotic cells using either fusion proteins [10], [11] or multiple plasmids [12] in the past two decades. Unfortunately, despite its success as a bacterial reporter, development of the bacterial luciferase as a reporter for widespread use, allowing real-time detection in eukaryotic cells, has faced difficulties, such as low expression levels, lack of thermostability, and poor quantum yield. These hurdles were partially overcome through codon-optimization, addition of specialized linker regions, and co-expression of the flavin reductase gene (*frp*) encoding the NADPH-FMN oxidoreductase [13], but substantial light production by the *lux* reaction was demonstrated to occur only in the lower eukaryote *Saccharomyces cerevisiae*. Recently, Close et al. [14] have reported a major advance in developing an autonomous mammalian light production system by expression of all five genes (*luxCDABE*) of the *lux* operon simultaneously in a mammalian background. However, it should be noted that the bioluminescent signal from the human-optimized *lux* cassette was also relatively weak (several orders of magnitude lower than that of the Fluc reporter). Thus, it is clear that the bacterial luciferase could potentially benefit from further optimization to reach its full potential as a eukaryotic reporter.

In this research, to simplify the usage of the bacterial luciferase as a reporter protein in eukaryotic cells, we have constructed a new fused *luxAB* gene encoding a fusion protein as a reporter. Furthermore, we show that the activity of the fused bacterial luciferase was significantly increased by several steps of directed evolution, compared to the wild-type fused *luxAB* enzyme. The enhanced *luxAB* fusion gene has been successfully expressed in protoplasts of *Arabidopsis* and maize. These results show promise toward the potential development of a eukaryotic reporter system allowing *in vivo* real-time detection in the future.

## Materials and Methods

### Bacterial strains and vectors

Bacterial strains and plasmids used in this study are listed in Table S1. *Escherichia coli* and *Corynebacterium glutamicum* strains were cultured in Luria-Bertani (LB) broth or on LB plates at 37°C and 30°C, respectively. *Yersinia pseudotuberculosis* strains were cultured in Yersinia-Luria-Bertani (YLB) broth (1% tryptone, 0.5% yeast extract, 0.5% NaCl) or on YLB plates at 26°C [15]. These bacterial strains represent a wide spectrum of possible prokaryotic organisms in which a *lux* reporter system might be useful. When needed, antibiotics were used at the following concentrations: ampicillin, 100 µg/ml for *E. coli* and *Y. pseudotuberculosis*; chloroamphenicol, 20 µg/ml for *E. coli* and 10 µg/ml for *C. glutamicum*; kanamycin, 50 µg/ml for *E. coli* and *Y. pseudotuberculosis*, and 25 µg/ml for *C. glutamicum*; nalidixic acid, 30 µg/ml for *C. glutamicum.*

The enhanced *luxAB* reporter was also tested in eukaryotic cells represented by the common baker’s yeast *Saccharomyces cerevisiae* and in leaf protoplasts of *Arabidopsis thaliana* and *Zea mays* (maize). YPD liquid medium (1% yeast extract, 2% peptone, 2% glucose) was used for routine growth of yeast. Plasmid p425GPD used in this study contained the LEU2 selectable marker and a glyceraldehyde 3-phosphate dehydrogenase (GPD) promoter which controls the expression of exogenous proteins. *S. cerevisiae* strains harboring plasmids p425GPD were propagated in synthetic complete (SC) minimal media lacking leucine and containing 0.67% yeast nitrogen base (Invitrogen, Carlsbad, CA, USA) [16].

The method of protoplast preparation was optimized from previously published methodology [17]. Fully expanded leaves from 4 weeks old *Arabidopsis* (ecotype Columbia), growing under neutral or short photoperiods (12–13 h light or less) of 50–150 µE, were used to prepare protoplasts. Leaf strips (0.5 mm) were cut length-wise and vacuum infiltrated with digestion solution (1% cellulase, 0.2% macerozyme, 0.1% BSA, 0.035% β-mercaptoethanol, 10 mM CaCl_2_⋅2H_2_O, 0.4 M mannitol, 20 mM KCl, 20 mM MES, pH 5.7) for 30 min at −80 kPa pressure. The cells were then incubated on a rotary shaker (40 rpm) at 23°C for 1.5 h in the dark, followed by swirling at 80 rpm for 1 min. The digested cells were filtered through 75 µm mesh filters. The protoplasts were collected by centrifugation at 300 rpm for 5 min, washed once with digestion buffer, before being re-suspended with MMG solution (0.4 M mannitol; 15 mM MgCl_2_; 4 mM MES, pH 5.8).

For maize protoplast preparation as published [18], three day old, light-grown B_73_ seedings were moved to the dark at 25°C until the second leaf was about 10–15 cm long. The central 6–8 cm of the second leaf were cut into 0.5 mm strips length-wise and vacuum infiltrated with enzyme digestion solution (1.5% cellulase, 0.3% macerozyme, 0.1% BSA, 0.05% β-mercaptoethanol, 1 mM CaCl_2_⋅2H_2_O, 0.6 M mannitol, 10 mM KCl, 20 mM MES, pH 5.7) for 50 min with a pressure of −80 kPa. The digested cells were then incubated on a rotary shaker (40 rpm) at 25°C for 2 h in the dark, followed by swirling at 80 rpm for 5 min. Cells were then filtered through 45 µm mesh filters. Protoplast collection, washing, and resuspension were as described above for *Arabidopsis*.

### Construction of plasmids

Primers used in this study are listed in Table S2. The entire coding region of the cloned pDM4-*luxCDABE* was used as a template to amplify a fused *luxAB* gene by overlap PCR generated using the gene SOEing method described by Horton et al. [19]. In the first round of PCR, the 1083 bp *luxA* gene and the 984-bp *luxB* gene PCR products were amplified using primer pairs *lux*A-XbaI-F/*lux*A-L_15_-R and *lux*B-L_15_-F/*lux*B-BglII-R. The resulting PCR products were used as template in the second round of PCR with *lux*A-XbaI-F and *lux*B-BglII-R as primers. The 2.1 Kb PCR product was recovered from an agarose gel and digested with XbaI/BglII and then ligated into XbaI/BamHI digested pBluescript II KS(+). This construct was named pBS-*luxAB*. The *lac* promoter in plasmid pUT18 and T6SS4 promoter in the enteric pathogen *Y*. *pseudotuberculosis* [15] were amplified with primer pairs p*Lac*-SacI-F/p*Lac*-XbaI-R and p*T6SS4*-SacI-F/p*T6SS4*-XbaI-R, respectively, and digested with SacI/XbaI and ligated into the same restriction enzyme digested pBS-*luxAB* and pBluescript II KS(+), resulting in plasmids pBS-p*Lac*::*luxAB*, pBS-p*Lac*, pBS-p*T6SS4*::*luxAB* and pBS-p*T6SS4*. To produce the expression plasmids pET28a-*luxA+B* and pET28a-*luxAB*, the bicistronic *luxA+B* gene amplified from pDM4-*luxCDABE*, and the *luxAB* gene fusion amplified from pBS-p*Lac*::*luxAB* using the primers *lux*A-BamHI-F/*lux*B-SalI-R were cloned into compatible sites within the pET28a vector downstream of the T7 promoter, respectively.

The enhanced *luxAB* (e*luxAB*) generated by DNA shuffling described below was amplified using *lux*A-XbaI-F and *lux*B-BglII-R primers and digested with restriction enzymes XbaI/BglII and ligated into pBS-p*Lac* digested with XbaI and BamHI. After transformation and selection on plates with ampicillin, the plasmid pBS-p*Lac*::e*luxAB* was obtained. Similarly, the plasmid pXMJ19-p*Tac*::e*luxAB* was constructed by inserting the e*luxAB* gene downstream of the *tac* promoter in pXMJ19 plasmid with the unique restriction sites BamHI and SalI, and the plasmid pBS-p*T6SS4::*e*luxAB* was constructed by inserting the e*luxAB* gene downstream of the T6SS4 promoter in pBluescript II KS(+). Plasmids pBS-p*Lac*::e*luxAB*, pXMJ19-p*Tac*::e*luxAB* and pBS-p*T6SS4::eluxAB* were electroporated into *E. coli* DH5α, *C. glutamicum* RES167 and *Y. pseudotuberculosis* YPIII cells, respectively.

The *luxAB*, e*luxAB* and codon optimized e*luxAB* (opt-e*luxAB*) described below were introduced into the plasmids p425GPD with the restriction sites BamHI at the 5′ end and SalI at the 3′ end to produce the plasmid p425GPD-*luxAB*, p425GPD-e*luxAB* and p425GPD-opt-e*luxAB*. These three plasmids were introduced into *S. cerevisiae* strains and selected on SC minimal selective media.

The plant expression vector was constructed as follows: For plasmid a1 construction, the e*luxB* fragment was generated by PCR from pBS-*pLac*::e*luxAB* using a pair of primers eluxB-NcoI-F and eluxB-R, the *nos* fragment was PCR-amplified using a pair of primers nos-F and nos-SalI-R from the pGL3-dual luciferase vector [20], then the e*luxB-nos* fragment was amplified by overlapping extension PCR using a pair of primers eluxB-NcoI-F and nos-SalI-R and using mixed e*luxB* and *nos* fragments as templates. The PCR products were digested with NcoI and SalI and cloned into the corresponding sites of the dual luciferase vector to replace the Fluc ORF. The designed primers eluxA-NheI-F and eluxA-XbaI-R, were used to PCR-amplify the e*luxA* fragment from pBS-p*Lac::*e*luxAB*, after digested by NheI and XbaI, the fragment was cloned into the dual luciferase vector to replace another *Renilla* luciferase (Rluc) ORF. For plasmid a2 construction, the opt-e*luxB* fragment was amplified using primers opt-eluxB-NcoI-F and opt-eluxB-R, from the pUC57-Simple plasmid which includes the optimized sequence of e*luxAB*, the same strategy was applied to obtain the opt-e*luxB-nos* fragment, the primers used for amplification of opt-e*luxB-nos* were eluxB-NcoI-F and nos-SalI-R. The opt-e*luxA* fragment were amplified from pUC57-Simple plasmid by PCR-amplify using primers opt-eluxA-NheI-F and opt-eluxA-XbaI-R. The opt-e*luxA* fragment was cloned into a1 vector to replace the e*luxA* fragment. For plasmid a3 construction, the e*luxAB* fragment was amplified from pBS-p*Lac::*e*luxAB* using primers eluxA-NheI-F and eluxB-XbaI-R. The PCR products and the a1 vector were digested by NheI and XbaI and then purified by Cycle Pure Kit (OMEGA Co., Ltd). The two purified fragments were ligated by T4 DNA ligase. For plasmid a4 construction, the opt-e*luxAB* fragment was obtained by digestion of pUC57-Simple plasmid by NheI and XbaI restriction enzymes and then recycled and purified. The fragment was then cloned into plasmid a3 to replace the e*luxAB* fragment. For plasmid a5 construction, the single copy of the CaMV35S promoter was amplified from pCAMBIA3301 using primers 35S-KpnI-F and 35S-NheI-R, the single copy of the CaMV35S promoter was used to replace the double CaMV35S promoter in a4 vector.

### Overexpression and purification of recombinant proteins

Plasmids pET28a-*luxAB* or pET28a-*luxA+B* were transformed into *E. coli* BL21(DE3) and the recombinant bacteria containing these plasmids were grown at 37°C in LB medium to an OD_600_ of 0.5. The strains were then shifted to 20°C and induced with 0.4 mM isopropyl-β-D-thiogalactopyranoside (IPTG) and then cultivated for an additional 16 h at 20°C. Approximately 100 µl of culture was centrifuged, and the pellet was resuspended in 100 µl of 1×SDS sample buffer. The samples were heated at 98°C for 10 min and subjected to SDS-PAGE analysis on 12% gels made with 29∶1 acrylamide:bisacrylamide stock (Ameresco). Electrophoresis of 5 µl samples proceeded at 15 mA for about 1.5 h before staining with Coomassie blue with gentle rocking at RT for 2 h. Gels were destained in 10% acetic acid, 40% methanol at RT until the protein bands were clearly visible. For purification of 6×His-tagged fusion proteins, harvested cells were resuspended in lysis buffer (10 ml/g pellet) containing 50 mM Tris-HCl (pH 8.0), 100 mM NaCl, 0.5 mg/ml lysozyme, and 2 mM phenylmethylsulfonyl fluoride (PMSF) and then incubated on ice with occasional vortexing for 10 to 15 min until the suspension became viscous. The cells were broken by sonication on ice until the suspension was translucent. Cleared cell lysates were incubated with Ni^2+^ agarose beads for 2 h at 4°C, and the beads were washed with 40 times the bed volume of TBS buffer (50 mM Tris-HCl, 150 mM NaCl, pH 7.6) containing 10 mM imidazole. Recombinant 6×His-tagged LuxAB and LuxA+B proteins were eluted with TBS buffer containing 200 mM imidazole and exhaustively dialyzed against TBS to remove imidazole. The molecular weight and concentrations of the purified proteins were also analyzed using 12% SDS-PAGE gels [21].

### Random mutagenesis

To increase the luciferase activity of *luxAB*, random mutations were introduced with either three rounds of error-prone PCR or two rounds of chemical mutagenesis. For error-prone PCR, random mutations were introduced into the amplification product during PCR by using the plasmid pBS-p*Lac*::*luxAB* as template and oligos *lux*A-XbaI-F and *lux*B-BamHI-R as primers (Table S2). The mutagenesis frequency was controlled to the desired level (two to four amino acid substitutions per kb) by altering the concentration of the template and the cycle numbers. For a typical reaction, 0.2 ng template DNA were added to 100 µl of the error prone PCR system (0.2 mM of each dATP and dGTP, 1.0 mM of each dCTP and dTTP, 7 mM MgCl_2_, 50 mM KCl, 1 mM MnCl_2_, 10 mM Tris-HCl (pH 8.3), 0.3 µM of primer *lux*A-XbaI-F and *lux*B-BamHI-R, 5U Taq DNA polymerase) and program subjected to 25 PCR cycles [22], [23]. Chemical mutagenesis was performed by treating the pBS-p*Lac*::*luxAB* plasmid DNA with hydroxylamine. In this instance, 5 µg of plasmid DNA was incubated in a 100 µl reaction mixture containing 0.5 M hydroxylamine, 0.5 mM Na_2_EDTA, and 5 mM Tris-HCl (pH 6.0) for 10 h at 37°C. The DNA was precipitated and washed thoroughly with 70% ethanol, and redissolved in ddH_2_O [24], [25]. The mutated *luxAB* produced in both error-prone PCR and chemical mutagenesis were recovered by digestion with XbaI/BamHI, and ligated into pBS-p*Lac* digested with the same enzymes, resulting in the pBS-p*Lac*::luxAB* mutant library and was transformed into the *E. coli* strain DH5α for storage and further screening.

### DNA shuffling and screen assay

The substrates for the shuffling reaction were nine mutant plasmids (pBS-p*Lac*::luxAB*) generated from the prior error prone PCR or chemical mutagenesis that showed enhanced *lux* quantum yield. These nine templates were each used to amplify a 2.1-kb PCR product using primers *lux*A-XbaI-F and *lux*B-BamHI-R. After the amplicons were purified, about 4 µg was digested with 0.15 unit of DNase I (Promega) in 100 µl of 50 mM Tris-HCl (pH 7.4), 1 mM MgCl_2_ for 10–20 min at room temperature. The digestion was loaded on a 2% low melting point agarose gel and the smear from 50 to 100 bp was purified by electrophoresis onto DE81 ion-exchange paper (Whatman) and eluted with 1 M NaCl, then precipitated by ethanol. The purified fragments were redissolved in a PCR reaction mixture at a concentration of 30 ng/µl and no primers were added at this point. Taq DNA polymerase (TaKaRa, Dalian, China) was used at 2.5 units, and ddH_2_O added to a total volume of 100 µl and subjected to 40–45 PCR cycles. The PCR products were purified by gel extraction. After 1∶40 dilution of these primerless PCR products into a second PCR reaction mixture, 0.3 µM of each primer *lux*A-XbaI-F and *lux*B-BamHI-R were added and 15 additional PCR cycles were applied to typically obtain a single amplicon of the expected, 2.1 kb size [26], [27].

Plasmid pBS-p*Lac*::*luxAB* constructs from the random mutagenesis or subsequent DNase I recombination library, were transformed into *E. coli* DH5α cells and grown at 37°C to an OD_600_ of 0.8 in LB medium with ampicillin (100 µg/ml). 200 µl of each culture was resuspended and placed into a well of a 96-well black plate, before the rapid addition of 2 µl decanal in each well [28]. The *lux* activity of these assays was estimated with an Infinite M200 PRO (TECAN). Strains which had the greatest luminescence activity, relative to the original construct, were called e*luxAB* (enhanced *luxAB*) and served as templates for the next experiment.

### Effects of pH and temperature on enhanced luciferase activity

The optimum pH of the best e*luxAB* enzyme’s activity was determined within a pH range of 4–12. The buffers were as followed: acetate (pH 4–5); phosphate (pH 6–8); glycine-NaOH (pH 9–12) [29]. The pH optimum was measured by incubating the cells in different buffers in the pH range of 4–12 at 37°C using decanal as substrate. The effect of temperature on enzyme activity was measured by incubating the enzyme from 20 to 60°C for 30 min at the previously determined pH optimum of 10. The activities of e*luxAB* were measured under standard assay conditions.

### Complete codon optimization of e*luxAB*

The e*luxAB* sequence was codon optimized according to *Arabidopsis thaliana* codon usage data from the information tabulated in GenBank. The overall ratio for usage of each codon within the e*luxAB* gene was altered to more closely match *Arabidopsis* usage. The OptimumGene™ algorithm optimized a variety of parameters critical to the efficiency of gene expression, including but not limited to codon usage bias adjustment; GC content adjustment; restriction enzymes and CIS-acting elements; removal of repeat sequences and mRNA secondary structure mitigation. All efforts focused on defining a single gene sequence that would attain the highest possible level of expression in the plant cell. Once optimized, the genes were synthesized by GenScript Co., Ltd (Nanjing, China) and polyacrylamide gel (PAGE) purified to ensure full-length products. These optimized DNA fragments were ligated using TA cloning and the sequence was verified in each case [14], [30]. The codon optimized e*luxAB* was named opt-e*luxAB* and the optimized bicistronic e*luxA+B* was named opt-e*luxA+B*, respectively.

### PEG mediated transformation of *Arabidopsis* and maize protoplasts

PEG-Ca mediated transformation followed a previously published protocol [17] with some modifications. The protoplasts of *Arabidopsis* were suspended in 100 µl of MMG solution at a density of 10^7^ (and the maize protoplasts at 5×10^6^) cells/ml in a 2 ml round-bottom centrifuge tube containing 15 µg of DNA and mixed well. An equal volume of 40% PEG solution (40% PEG4000, 0.2 M mannitol and 100 mM CaCl_2_⋅2H_2_O) was added to the protoplast-DNA mix drop-wise with gentle shaking for 25 min. The mixture was diluted with 440 µl of cell culture solution which, depending on the species, was: (*Arabidopsis*: 154 mM NaCl, 125 mM CaCl_2_⋅2H_2_O, 5 mM KCl, 2 mM MES pH5.7) or (maize: 0.6 M mannitol, 4 mM KCl, 4 mM MES, pH 5.7). The protoplasts were then centrifuged at 112×g, the supernatant removed, the protoplasts re-suspended in 1 ml cell culture solution, and plated in the wells of 24-well tissue culture plates (NEST Biotechnology, China). The protoplasts were incubated at 23°C (*Arabidopsis*) or 25°C (maize) for 18 h prior to harvest for luciferase activity determination.

### Luciferase activity detection

*E. coli* cells transformed with pBS-p*Lac*::*luxAB* or pBS-p*Lac*::e*luxAB* constructs were grown at 37°C to an OD_600_ of 1.0 in LB broth with ampicillin (100 µg/ml). *C. glutamicum* cells transformed with pXMJ19-p*Tac*::*luxAB* or pXMJ19-p*Tac*::e*luxAB* constructs were grown at 30°C to an OD_600_ of 1.8 in LB broth with chloramphenicol (20 µg/ml). *Y. pseudotuberculosis* cells transformed with pBS-p*T6SS*::*luxAB* or pBS-p*T6SS*::e*luxAB* were grown at 26°C to an OD_600_ of 0.8 in YLB medium with ampicillin (100 µg/ml). Yeast cells containing plasmids p425GPD-*luxAB* and p425GPD-e*luxAB* were grown at 30°C to an OD_600_ of 1.0 in synthetic complete (SC) minimal medium. 100 µl of each resuspended culture was transferred into a 1.5 ml microcentrifuge tube and luminescence reactions were initiated by the addition of decanal. The activity determinations of the fused *luxAB* and enhanced mutants were performed on the GloMax 20/20 Luminometer (Promega) as described [31]. Protoplasts from one well of a 24 well plate were recovered by aspiration, placed in an Eppendorf tube and harvested by centrifugation at 1300 rpm for 5 min before being resuspended in 20 µl of lysis buffer (100 mM potassium phosphate, 1 mM dithiothreitol, pH 7.8). The cells were subjected to three rounds of freezing in liquid nitrogen for 30 sec, followed by thawing in a 37°C water bath for 3 min as described [14]. 200 µl detection solution (0.2 mM NAD(P)H, 80 µM FMNNa, 1% (v/v) decanal) was added, vortexed, and the solution assessed for luciferase activity. After the substrate was added, the luminescence activity was measured.

### Statistical analysis

Statistical analysis was conducted using GraphPad Prism 5.01 software.

## Results

### Expression of the fused and non-fused *luxA* and *luxB* gene in bacteria

**Figure 1:**
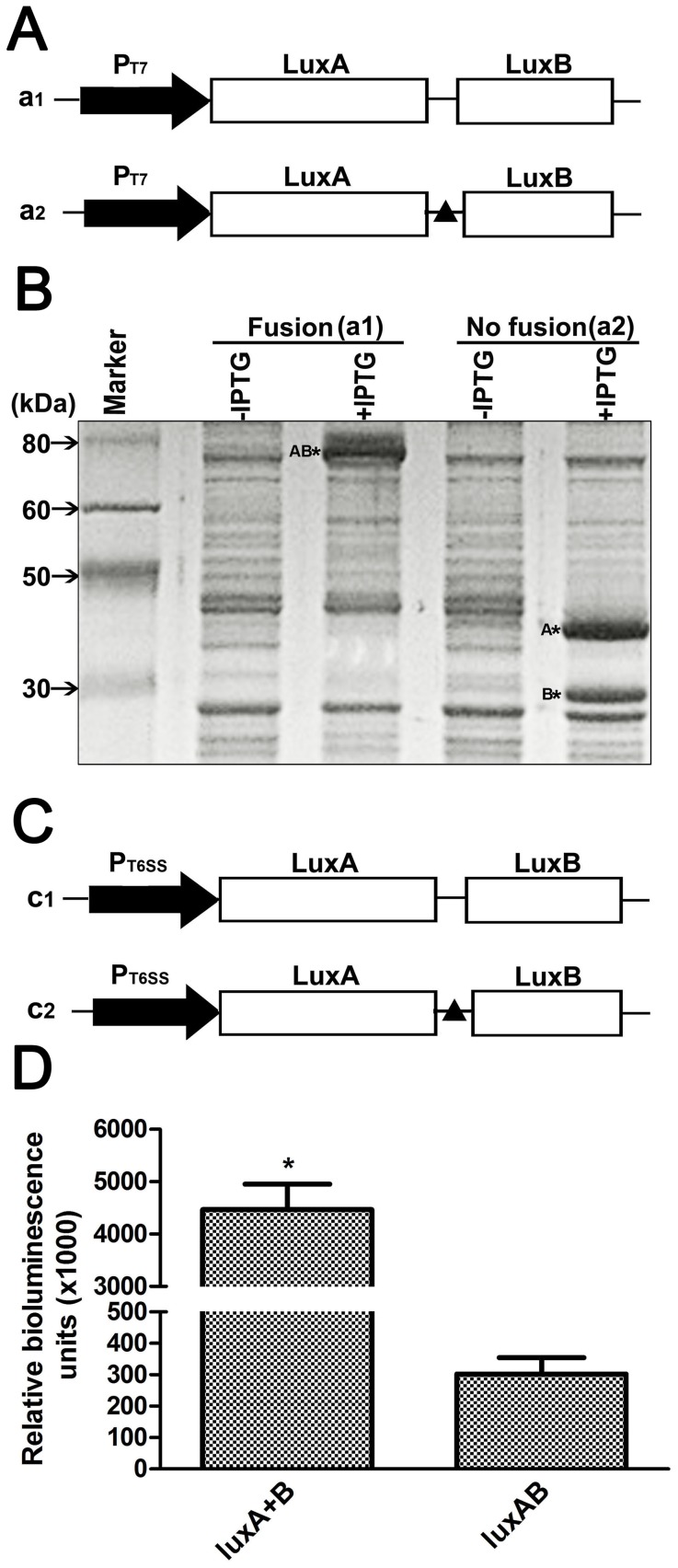
Expression of *lux*A and *lux*B genes in bacteria cells. **A.** Schematic representation of expression constructs of *lux*A and *lux*B genes in the pET28a vector. The *lux*A and *lux*B genes either fused in one cistron or in two separate cistrons, were under the control of the T7 promoter. a1: Fusion construct of *lux*A and *lux*B genes. The ORFs of *lux*A and *lux*B were fused using a 15 amino acid linker (GGGSG)_3_ and the stop condon of the *lux*A ORF was removed. a2: the *lux*A and *lux*B ORFs were separated by an intergenic sequence and were translated independently. **B.** The constructs in (**A**) were expressed in BL21(DE3) cells. Protein expression was induced by IPTG. The cell lysate was separated by SDS-PAGE and the gel subjected to Coomassie blue staining. **C.** Schematic representation of expression constructs of *lux*A and *lux*B genes in the pBS-p*T6SS4* vector. The constructs are similar as described in (**A**) except that the *lux*A and *lux*B genes were under the *T6SS4* promoter. **D.** Comparison of luciferase activity from the LuxAB fusion protein or the separate LuxA and LuxB heterodimeric proteins in *Y. pseudotuberculosis*. The luminescence reaction was initiated by the addition of 1% decanal as substrate. Data are means±se, n = 3. *P<0.01.

Cell extracts from *E. coli* BL21(DE3) harboring pET28a-*luxAB* possessed a 78-kDa *luxAB* fusion protein clearly present in IPTG induced bacteria when compared with the non-induced lane (Figs. 1A&B). Extracts from induced BL21(DE3) cells possessing the non-fused pET28a-*luxA+B* construct show a 40 kDa α subunit and a 36 kD β subunit polypeptide observed in the SDS-PAGE gel (Figs. 1A&B). The *luxAB* and *luxA+B* genes expressed under the *T6SS4* promoter in *Y. pseudotuberculosis* cells demonstrated that the luminescence intensity of *luxAB* is about 0.002% that of the *luxA+B* (Figs. 1C&D). These data indicate a potential of improving the *luxAB* activity by random mutagenesis and directed evolution.

### Error-prone PCR, chemical mutagenesis and DNA shuffling

**Figure 2:**
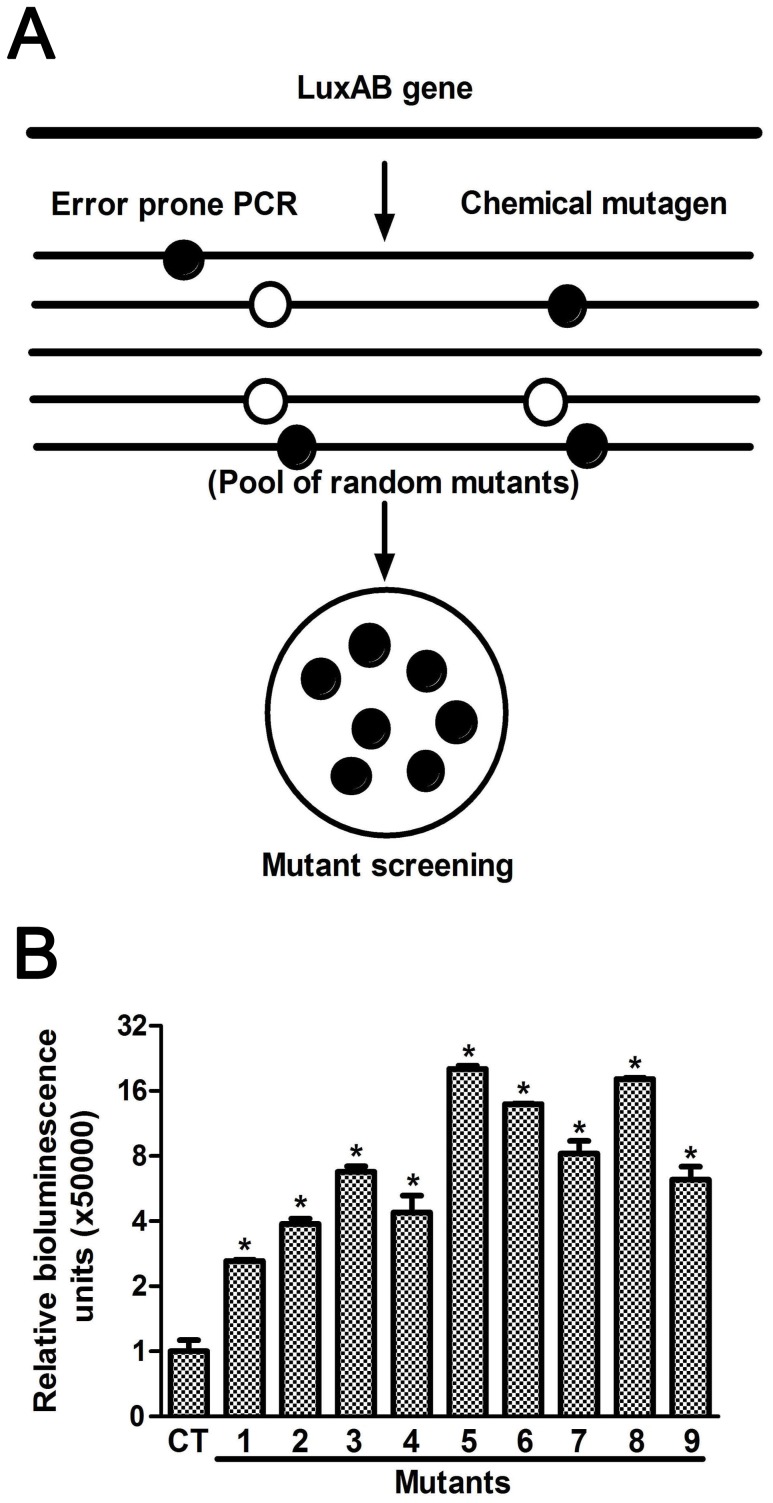
Generation of *luxAB* mutants with greater luciferase activity. **A.** Experimental strategy for generation of *luxAB* mutants with increased luciferase activity. The *luxAB* gene was mutated by error prone PCR and chemical mutagenesis. Closed circles indicate positive mutations; open circles indicate negative mutations. **B.** Relative luciferase activity of the positive mutants. The luciferase activity of mutants was normalized to the wild type control. Data are means±se, n = 3. *P<0.01 *vs* wild type control.

**Figure 3:**
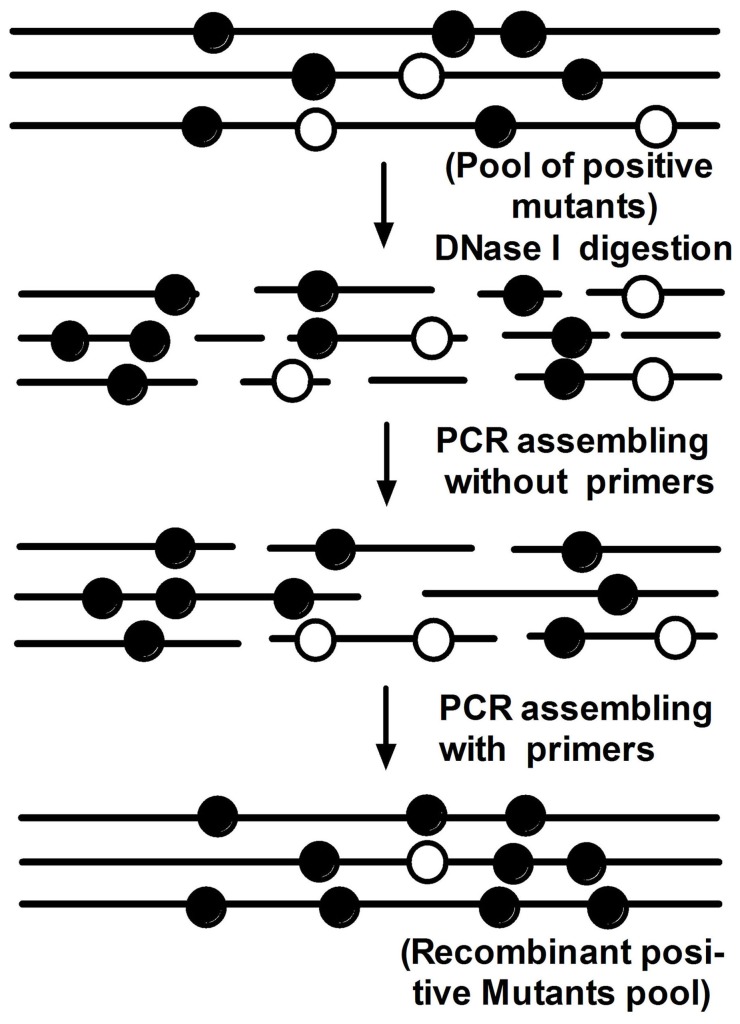
Generation of recombinant *luxAB* with increased luciferase activity. Experimental strategy of DNA shuffling of *luxAB* of the nine positive mutants. Closed circles indicate positive mutations; open circles indicate negative mutations.

Following mutagenesis, nine *luxAB* mutants were picked that had been individually verified to result in higher luciferase activity than the original *luxAB* (Figs. 2A&B). These genes, carrying positive mutations, were selected as a pool to initiate DNA shuffling. After DNase I digestion, 50 to 100 bp fragments were recovered to ensure the best recombination frequency. The *luxAB* mutants were then re-assembled by PCR (Fig. 3). After two rounds of DNA shuffling, about two thousand mutant colonies were screened. One mutant was identified (e*luxAB*, enhanced *luxAB*) with remarkably greater *lux* activity than all others tested. Compared to the sequence of wild type *luxAB*, there were six mutation sites in the e*luxAB* gene, five of which resulted in amino acid changes (Table 1). Of these five, four were in the *luxA* subunit.

**Table 1:** DNA and amino acid substitutions in e*luxAB* gene.

Position	Base substitution	Position in codon	Amino acid	Amino acid substitution
432	G→A	3	144	M→I
700	C→T	1	234	H→Y
967	A→G	1	323	I→V
1009	A→G	1	337	I→V
1318	T→C	1	440	E→K
1450	G→A	1	484	L→L

### Protein modeling

**Figure 4:**
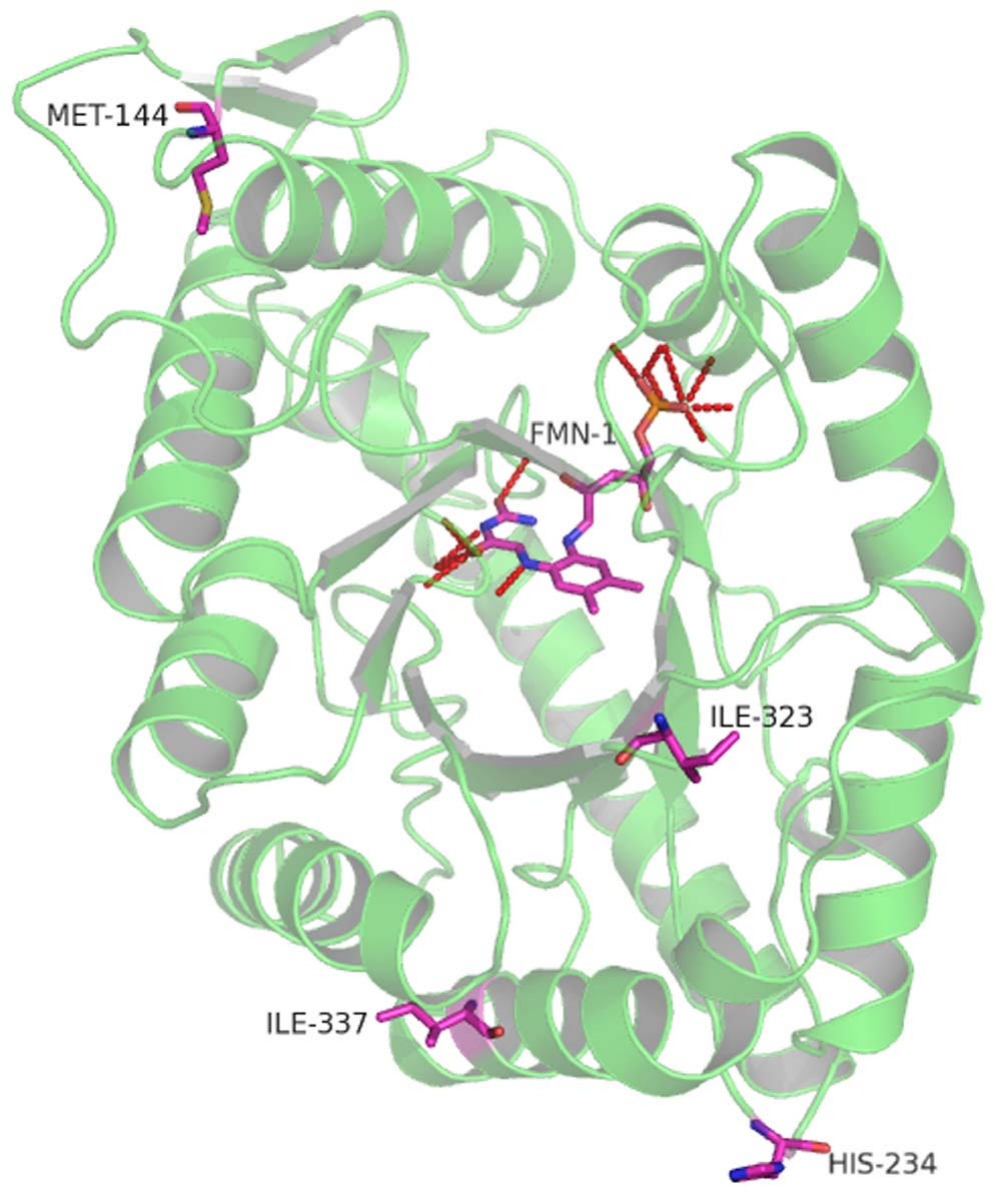
Positions of 4 different amino acid substitutions in e*luxAB* imposed on the structure of *luxA+B* from *V. harveyi* relative to the position of the flavin mononucleotide binding pocket (FMD).

The crystal structure of the *Vibrio harveyi* luciferase *luxA* and *luxB* holoenzyme has been resolved to 1.5 Å (1 Å = 0.1 nm) under low salt conditions [32]. Comparison of sequences showed that LuxA and LuxB from *P. luminescens* shared 84% and 59% amino acid identities with LuxA and LuxB from *V. harveyi*, respectively (Fig. S1). To investigate why the observed mutations of e*luxAB* enhanced luciferase activity, homology-based model structures of *luxA* and *luxB* from *P. luminescens* were obtained using structures of *V. harveyi* luciferase using the Swiss protein-modeling server [33]. Based on the model structures, none of the mutations directly affect the active site (FMN binding sites) or substrate binding residues (residues 166–233) [23]. However, the substitution of H234Y (Fig. 4) was in close proximity to the substrate-binding pockets and might indirectly affect the binding of the substrate. While the other mutation sites (Table 1) probably had no effect or only subtly changed the conformation of the active site and/or substrate specificity.

### Characterization of the e*luxAB*

**Figure 5:**
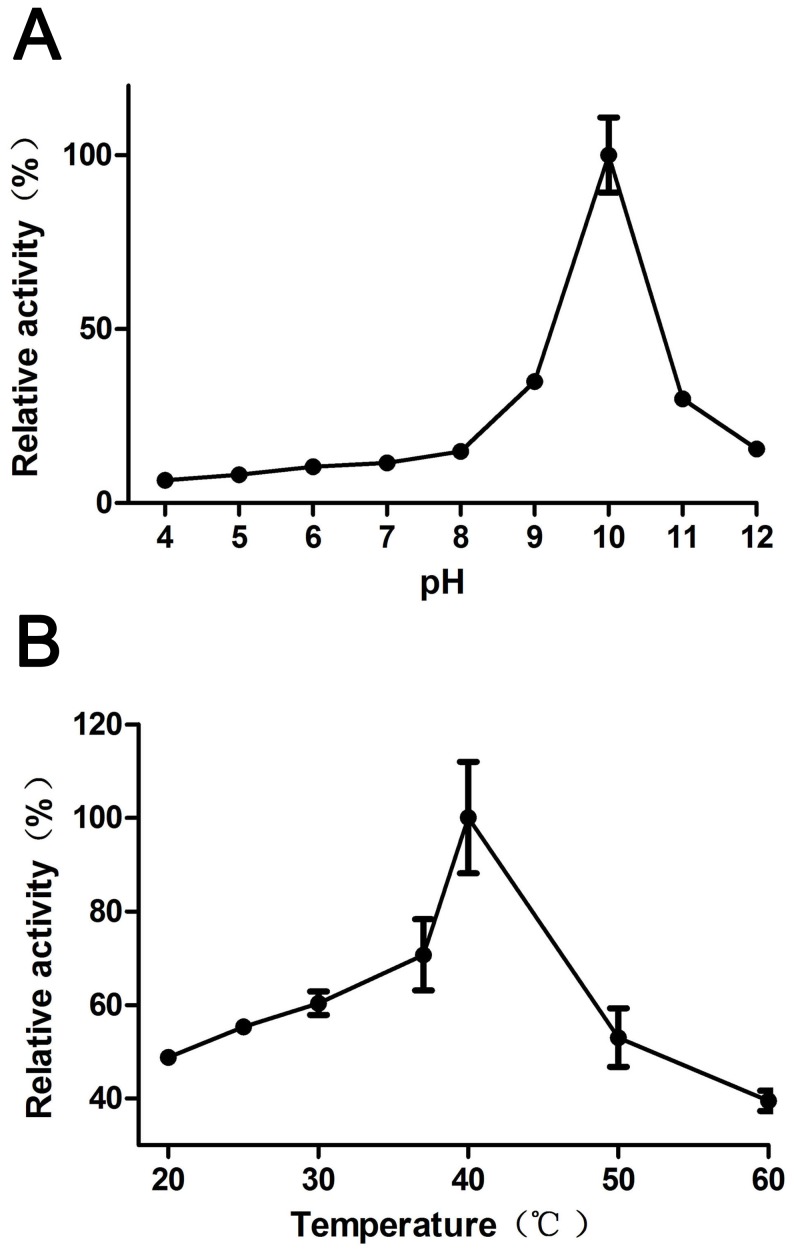
The pH (A) and temperature (B) optimum of eLuxAB in *Y. pseudotuberculosis*.

The optimal pH for e*luxAB* activity was distinctly centered around 10 using decanal as a substrate, with a rapid decline on either side of this value (the enzyme exhibited only 40% activity at pH 9.0 and 11.0; Fig. 5A). The temperature optimum of e*luxAB* at pH 10 was 40°C (Fig. 5B). Considerable activity was retained also at 30°C and 50°C after an incubation time of 30 min after which time residual activities were reduced to approximately 70% and 50%, respectively. Studies on the effect of pH and thermostability on the e*luxAB* clearly indicated that this enzyme is the best candidate for a wide range of applications where alkaline conditions prevail.

### Use of e*luxAB* as a reporter in *E. coli*, *C. glutamicum*, *Y. pseudotuberculosis* and Yeast cells

**Figure 6:**
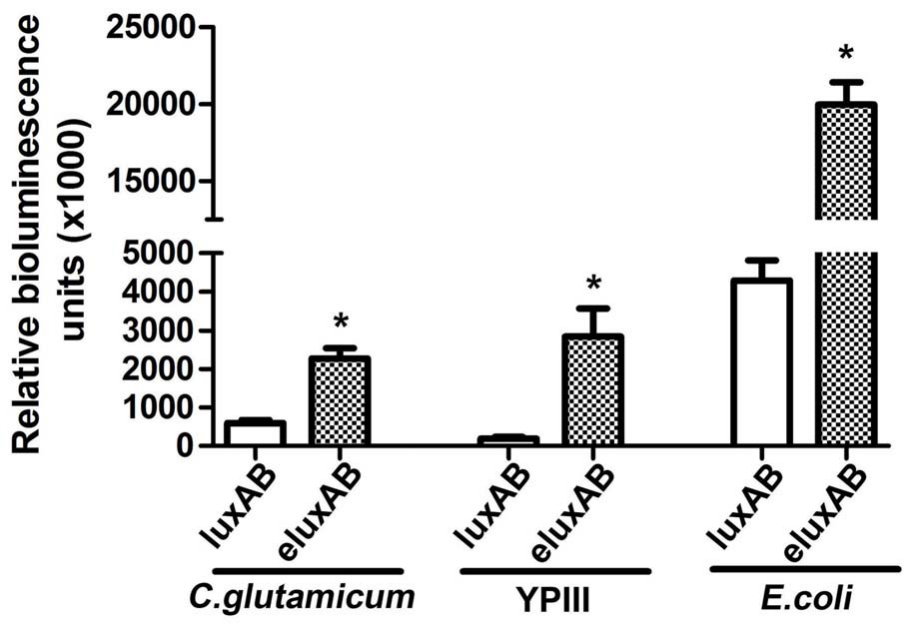
Relative luciferase activity of the engineered e*luxAB* gene in *C. glutamicum* RES167, *Y. pseudotuberculosis* YPIII and *E. coli*. Data are means±se, n = 3. *P<0.01 *vs* wild type control.

**Figure 7:**
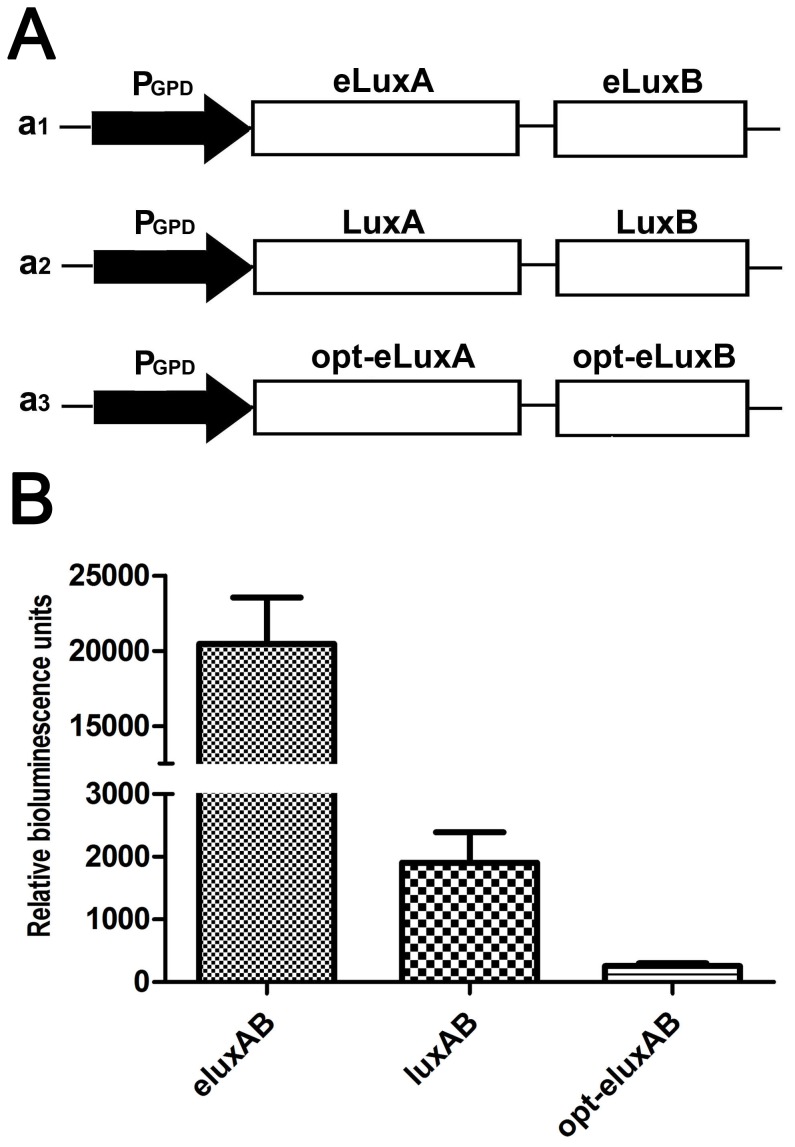
The application of the bacteria luciferase *luxAB* gene as a reporter tested in *S. cerevisiae* under the control of the GPD promoter. **A.** Schematic representation of expression vectors for *luxAB*, e*luxAB*, and opt-e*luxAB* gene fusions in yeast cells. **B.** Quantum yield of LuxAB, eLuxAB and opt-eLuxAB in yeast.

The *luxAB* and e*luxAB* were constructed in pXMJ19-p*Tac,* pBS-p*Lac*, pBS-p*T6SS4*, and p425GPD vectors under the control of their respective promoters. The plasmids pXMJ19-p*Tac*::*luxAB* and pXMJ19-p*Tac*::e*luxAB*, when expressed in the Gram-positive bacterium *C. glutamicum* resulted in an e*luxAB* luminescence approximately 4-fold greater than that of *luxAB* (Fig. 6). Similarly, the plasmids pBS-p*Lac*::*luxAB* and pBS-p*Lac*::e*luxAB* were transformed into *E. coli* cells and the luminescence was detected. Enzyme activity of e*luxAB* is about five-fold higher than *luxAB* (Fig. 6). The greatest increase in luminescence was for *Y. pseudotuberculosis* cells where pBS-p*T6SS4*::e*luxAB* registered a quantum yield 40 fold greater than that of pBS-p*T6SS4*::*luxAB* (Fig. 6). *Saccharomyces cerevisiae* cells harboring p425GPD-*luxAB* or p425GPD-e*luxAB* had an e*luxAB* activity approaching 10-fold that of *luxAB* (Figs. 7A&B). Thus we conclude that e*luxAB* can be used as a robust reporter in a variety of prokaryotic and simple eukaryotic species.

### Codon optimization of the e*luxAB* gene for *Arabidopsis* expression and use of opt-e*luxAB* as a reporter in *Arabidopsis* and maize protoplasts

**Figure 8:**
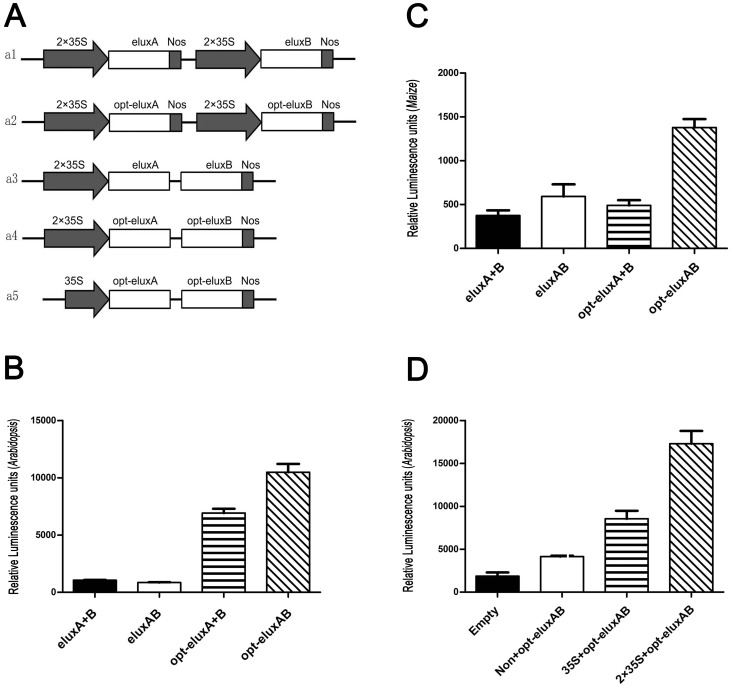
The application of the bacteria luciferase *luxAB* gene as a reporter in plant protoplasts. **A.** Schematic representation of expression vectors for e*luxAB*, or opt-e*luxAB* gene fusions in plant protoplasts driven by one or two copies of the CaMV35S promoter. **B.** Comparison of luciferase activity of eLuxA+B, eLuxAB, opt-eLuxA+B and opt-eLuxAB in *Arabidopsis*. **C.** Comparison of luciferase activity of eLuxA+B, eLuxAB, opt-eLuxA+B and opt-eLuxAB in maize. **D.** Comparison of opt-eLuxAB luminescence under different promoter strengths.

In order to maximize the expression of e*luxAB* in *Arabidopsis* and maize protoplasts, the codons of e*luxAB* were optimized according to *Arabidopsis* codon bias (Fig. S2). The optimized e*luxAB* gene opt-e*luxAB* (Genbank accession number: KJ957766) was also transformed into *S. cerevisiae* cells in vector p425GPD. Surprisingly, compared to the luciferase activity of e*luxAB* and *luxAB* in yeast, the quantum yield of opt-e*luxAB* was least (Fig. 7B). The possible reason was that *Arabidopsis* codon biased opt-e*luxAB* wasn’t suitable for expression in yeast cells. The monocistronic e*luxAB*, opt-e*luxAB* and the bicistronic e*luxA+B*, opt-e*luxA+B* were constructed in the pGL3 basic vector under the control of two copies of the CaVM35S promoter (Fig. 8A). The monocistronic opt-e*luxAB* cassette was also placed under the control of a single copy of the CaMV35S promoter. The plant expression vectors were transformed into *Arabidopsis* and maize protoplasts using PEG and, following incubation, the luminescence was detected. Compared to the non-codon-optimized e*luxA+B* or e*luxAB* genes, the opt-e*luxA+B* or opt-e*luxAB* showed dramatically increased luminescence activity in *Arabidopsis* cells (Fig. 8B). When these mono- or bi-cistronic e*luxAB* or opt-e*luxAB* vectors were tested in maize protoplasts, opt-e*luxAB* luminescence was approximately 3 fold greater than that of the other three constructs (Fig. 8C) although it was 6 times less than the same construct in *Arabidopsis* protoplasts (compare Figs. 8B&C) for which it was designed. To further determine if opt-e*luxAB* could be applied as a reporter gene in *Arabidopsis* cells, the opt-e*luxAB* gene was driven by one copy or two copies of 35S promoter and transformed into *Arabidopsis* protoplasts. The luminescence of opt-e*luxAB* under the regulation of two copies of 35S promoter was significantly greater than that produced when under the control of one copy of the 35S promoter (Fig. 8D).

To compare the luminescence activity of opt-e*luxAB* with Rluc or Fluc reporters, luminescence activities were compared in *Arabidopsi*s and maize protoplast cells transformed with Rluc and Fluc reporter vector (pGL3-dual luciferase) or opt-e*luxAB* reporter vector (pGL3-opt-e*luxAB*). The luminescence activity of opt-e*luxAB* is comparable to Rluc, though it is significantly lower than Fluc (Table S3). This result indicates that the bacterial luciferase has the potential to be applied as a new reporter in plant cells. Since the toxicity of decanal to *Arabidopsis* protoplast cells was negligible at the detection concentration (1%, Fig. S3), the opt-e*luxAB* reporter can be used for real-time detection in viable plant protoplast cells.

## Discussion

Engineering of an enhanced, monomeric bacterial luciferase e*luxAB* gene from *P. luminescens* as a reporter in prokaryotes and eukaryotes provides researchers a unique tool allowing real-time monitoring of exogenous gene expression from whole cells without lysis. A fused *luxAB* gene from *Photorhabdus luminescens* has been cloned and successfully expressed in yeast [16] and mammalian cells [14], [30] previously, but the luminescence intensity was limited, despite exogenous substrate addition or codon-optimization [9], [16]. In order to overcome these problems and develop the bacterial luciferase as a convenient reporter, here we demonstrate for the first time that the fused *luxAB* gene could be directed evolved using a series of random mutations and shuffling techniques to produce an e*luxAB* with increased luciferase activity in prokaryotic cells. Testing for enhanced *luxAB* activity in prokaryotic systems permitted rapid evaluation of the efficacy of thousands of mutants. Subsequently, the best of these mutants was successfully expressed as a codon-optimized form of the e*luxAB* gene in *Arabidopsis* and maize protoplasts. The e*luxAB* gene produced by shuffling has several advantages. First, the fused e*luxAB* derived from *P. luminescens* is easy to construct and convenient to detect due to the facile application of exogenous substrate (decanal), which easily permeates into the cells [8] and does not require cell lysis for e*luxAB* detection in prokaryotes and yeast cells [11]. Upon addition of the aldehyde substrate, the activity of e*luxAB* can be followed *in vivo* by measuring light emission [14], [31]. In contrast, bioreporters constructed using *lacZ*, *cat*, or Fluc, for example, require a succession of pre-processing steps involving cell lysis, permeabilization and/or the addition of secondary substrates before final signal generation [34], [35]. Second, compared to the reporter GFP, e*luxAB* could be measured without the need to generate, and yet exclude, excitation wavelengths, avoiding some problems such as photonic bleaching and high background [36], [37]. Third, previous work with *lux* genes isolated from *P. luminescens* has demonstrated that the luciferase is thermostable at temperatures as high as 45°C [38], which is a greater thermal stability than that of the *V. harveyi* or *V. fischeri* luciferase enzymes. Therefore, the bacterial luciferase system seems more suitable to the study of environmental or developmental changes in gene expression. High activity at alkaline pH is normally regarded as an appreciable character for the industrial production [29].

Directed evolution has become a powerful strategy for improving the activity of enzymes in a targeted manner coupling various methods such as error-prone PCR and chemical mutagenesis to generate large variant libraries [39]. In this study random mutagenesis and DNA shuffling of *luxAB* provides a directed approach to improve the activity of the fused bacterial luciferase. As a result, the e*luxAB* strain was produced, which shows considerably improved luciferase activity in a variety of organisms. There are five mutations resulting in amino acid substitutions and one silent mutation that occurred to result in e*luxAB*. Previous work on the luciferase enzyme determined that the *lux* catalytic properties were primarily determined by the *luxA* subunit [40]. In this paper, of the five missense mutations, four appeared in *luxA*. None of the mutations occurs in the catalytic active site or appears to directly affect substrate binding. Because the amino acid sequences of *luxA* and *luxB* are highly conserved according to the result of alignments of amino acid sequences of *luxA* and *luxB* from *P. luminescens* and *V. harveyi* (Fig. S1), it is likely that if mutations occurred in the active site, they would inactivate the luciferase. Therefore, although it is speculative, it is most probable that the mechanism resulting in enhanced *lux* activity involves mutations that exert their effects by indirectly changing the conformation of the aldehyde binding sites rather than by interacting with substrates directly.

The bacterial luciferase e*luxAB* can be assayed with high sensitivity compared to the *luxAB* both in intact bacterial- and yeast-cells. In addition, the opt-e*luxAB* or opt-e*luxA+B* has a greater luciferase activity than the e*luxAB* and e*luxA+B* in *Arabidopsis* cells. Evidently, codon optimization of e*luxAB* led to an increased luminescence. Greater luminescence may be caused by greater *lux* expression levels, greater luciferase activity due to superior folding of the tethered *luxA* and *luxB* subunits, higher quantum yield per catalytic round, or any of a plethora of different reasons or combinations thereof. The luciferase activity of monocistronic opt-e*luxAB* is greater than that of opt-e*luxA* and opt*-eluxB* expressed in two separate cistrons when expressed from the same promoter (Figs. 8B&C). It is tempting to speculate that the separate subunits cannot be assembled efficiently into a functional holoenzyme in *Arabidopsis* cells. What’s more, under the regulation of the 2×35S promoter, the luminescence of opt-e*luxAB* is about two times greater than that from one copy of the 35S promoter. This is compelling evidence that the opt-e*luxAB* can eventually be developed as a useful marker gene for the quantitative assessment and detection of different activities from promoters in transgenic plants.

The e*luxAB* is detectable using currently available technologies and offers prolonged expression without cell lysis in both prokaryotes and yeast [11]. The *lux* system derived from bacteria is particularly useful as a prokaryotic reporter because the *luxC*, *luxD*, and *luxE* genes can provide continuous supplies of the aldehyde substrate of *lux* within the cells without any external manipulations [14]. Of course, endogenous *luxA* and *luxB* genes within this operon may provide background *lux* readings precluding its use in these systems. However, modifications of the reporter system to include the other genes within the bacterial *lux* operon in the plant of interest may produce greater *lux* activity.

The plant-adapted bacterial luciferase gene opt-e*luxAB* also has some disadvantages. For example plant cells do not contain sufficient FMNH_2_ to drive the opt-e*luxAB* catalyzed reaction at *Vmax*, because FMN is enzyme-bound or enclosed in cell compartments in plant cells [34]. However, this is not a serious concern because, upon exogenous application of FMNH_2_, plant cells expressing *luxAB* may produce greater quantum yields [34], [41]. However, our ultimate goal is to have efficient expression and activity without the need of cell lysis or exogenous substrate application. One possible means to circumvent the poor availability of FMNH_2_ in the cytoplasm will be to target the opt-e*luxAB* into an organelle containing this cofactor using a target peptide on the N-terminus of opt-e*luxAB*. Further studies are needed to determine the best means by which to express the *frp* gene *in vivo* to produce enough FMNH_2_ in plant cells. Additionally, the basic pH optimum (10) of the e*luxAB* enzyme, and the abrupt attenuation of e*luxAB* activity to either side of this optimum is a major hurdle for the current use of this system in eukaryotic cells. Reducing the pH optimum of the e*luxAB* enzyme closer to that of the neutral pH of the cell is the current major focus of our synthetic biology approach using similar techniques to those described here. Alterations of pH optima and/or a broadening of the pH optimum, of several pH units, using directed evolution has been possible for a number of commercial applications [42], [43].

In a word, the e*luxAB* and opt-e*luxAB* demonstrate that, using directed evolution, synthetic biology, and by paying attention to codon bias, we can greatly increase the luminescence of a bacterial luciferase in plant cells, thus making it more suitable and convenient to use in these eukaryotes. This demonstration provides the foundation for further alterations in the opt-e*luxAB* gene to produce a reporter suitable as a practical bioprocess monitor, as a high-throughput promoter expression screening technique and in applications for medical diagnosis.

## Supporting Information

Figure S1 **Pairwise sequence alignment of the amino acid sequences between** ***luxA*** **and** ***luxB*** **genes from** ***P. luminescens*** **and** ***V. harveyi.***
(TIF)

Figure S2 **Alignment of e** ***luxAB*** **gene (Un-optimized) and codon-optimized e** ***luxAB*** **gene (Optimized) sequences.** Base changes are indicated in orange. The six mutation sites in the e*luxAB* gene are indicated with the black hollow box.
(TIF)

Figure S3 **The tolerance of** ***Arabidopsis*** **protoplasts to decanal treatment.** Protoplasts treated with different concentrations of decanal were photographed under optical microscope at time 0, 1 and 4 hours, respectively. Row 5 shows the protoplast cells tested with 200 µg/ml kanamycin as a control. Kanamycin but not decanal treatment leads to quick lysis of protoplast cells.
(TIF)

Table S1 **Bacterial strains and plasmids used in this study.**
(DOCX)

Table S2 **Primers used in this study.**
(DOC)

Table S3 **Comparison of luminescence activities between bacterial,** ***Renilla*** **and firefly luciferases in plant protoplasts.** Luminescence activities were measured in *Arabidopsis* and maize protoplast cells transformed with Rluc and Fluc reporter vector (pGL3-dual luciferase) or with opt-e*luxAB* reporter vector (pGL3-opt-e*luxAB*). The number of *Arabidopsis* protoplasts used was about 5×10^6^ and of the maize protoplasts was about 3×10^5^ in 100 µl volume culture.
(DOCX)
