# Supplementary figures and images for: Engineering an Enhanced, Thermostable, Monomeric Bacterial Luciferase Gene As a Reporter in Plant Protoplasts
Source: PLoS One. 2014 Oct 1;9(10):e107885. doi: 10.1371/journal.pone.0107885 (PMC4182741; doi:10.1371/journal.pone.0107885)

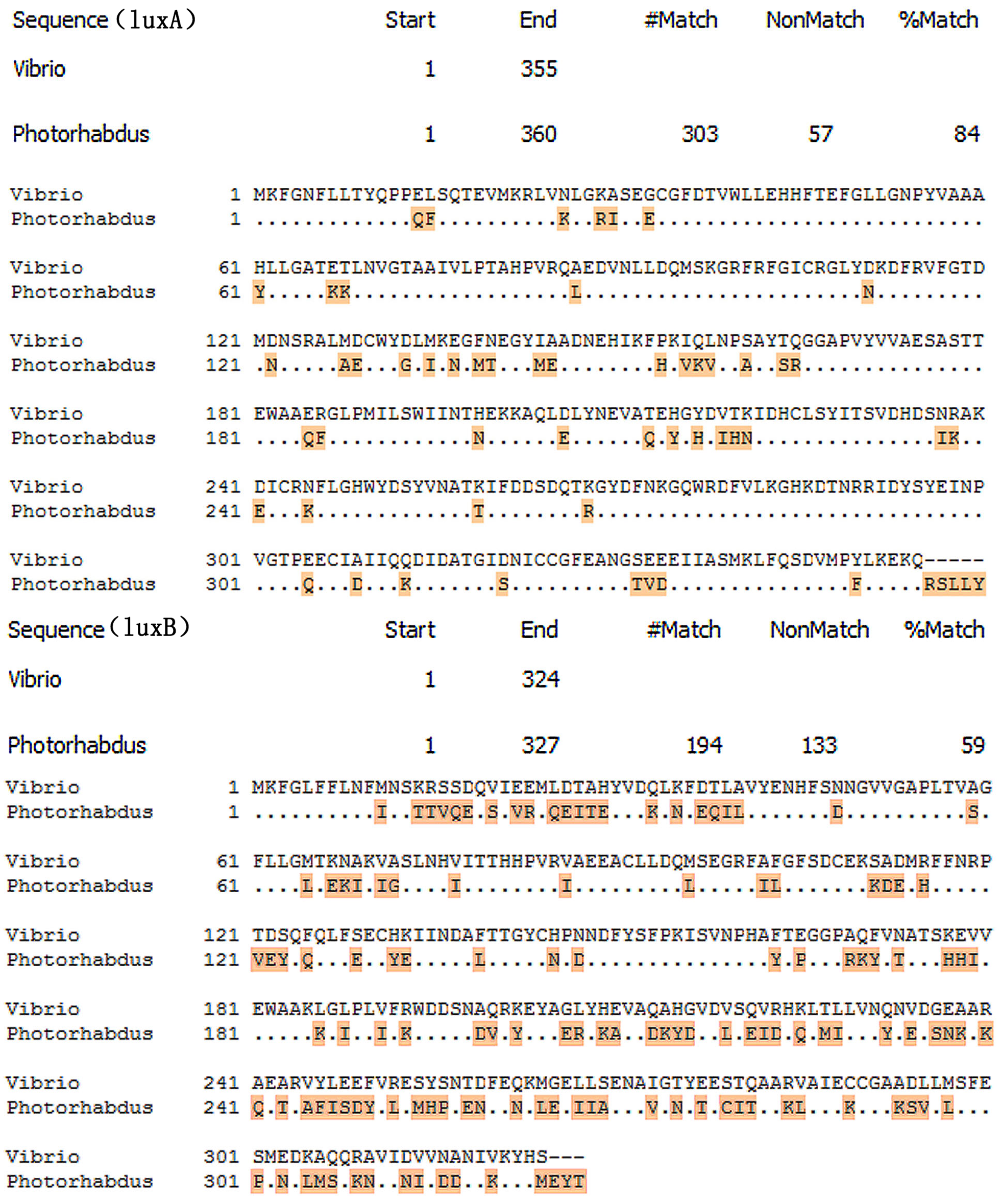

Supplement: Figure S1 — Pairwise sequence alignment of the amino acid sequences between luxA and luxB genes from P. luminescens and V. harveyi. (TIF) [file pone.0107885.s001.tif]

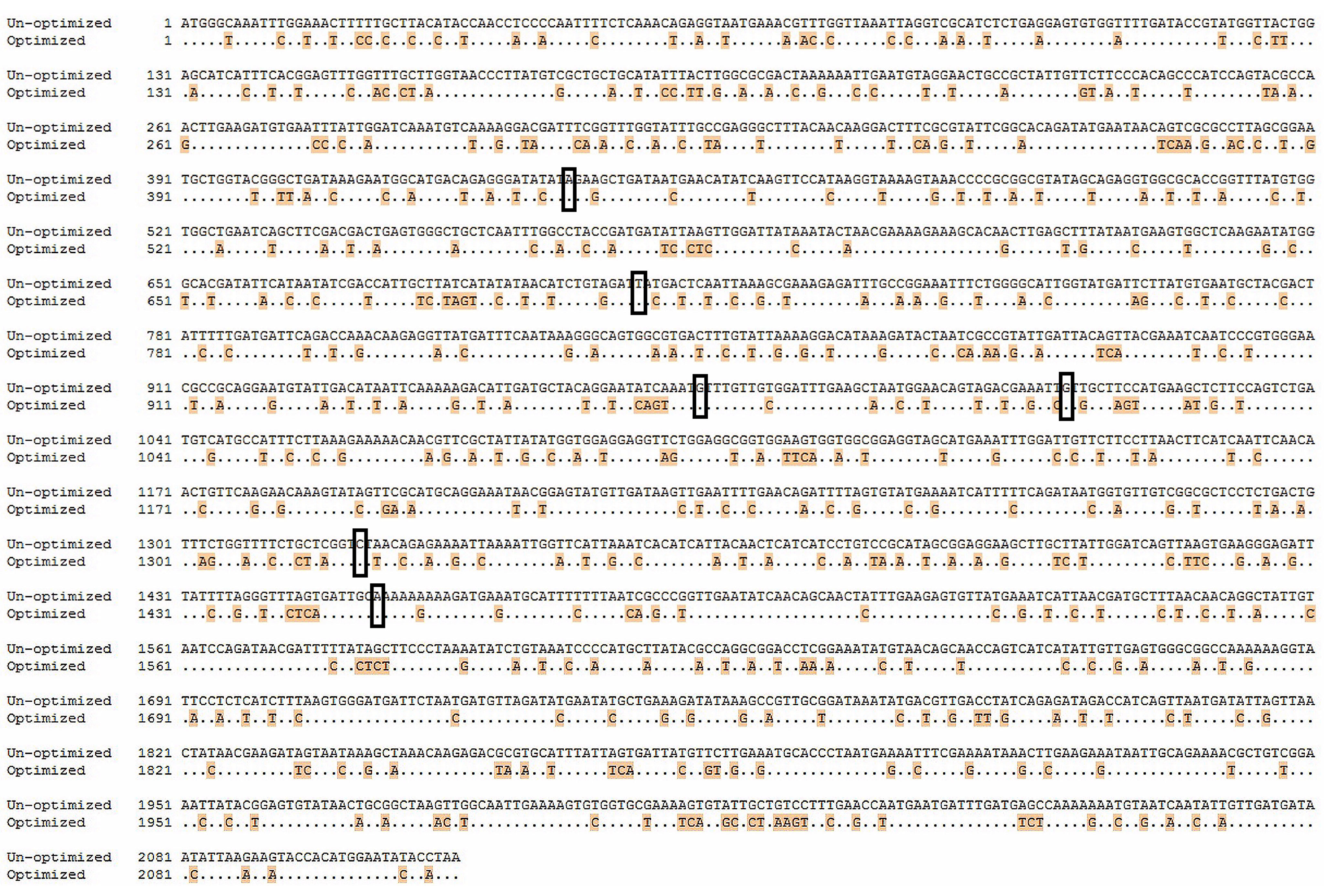

Supplement: Figure S2 — Alignment of e luxAB gene (Un-optimized) and codon-optimized e luxAB gene (Optimized) sequences. Base changes are indicated in orange. The six mutation sites in the eluxAB gene are indicated with the black hollow box. (TIF) [file pone.0107885.s002.tif]

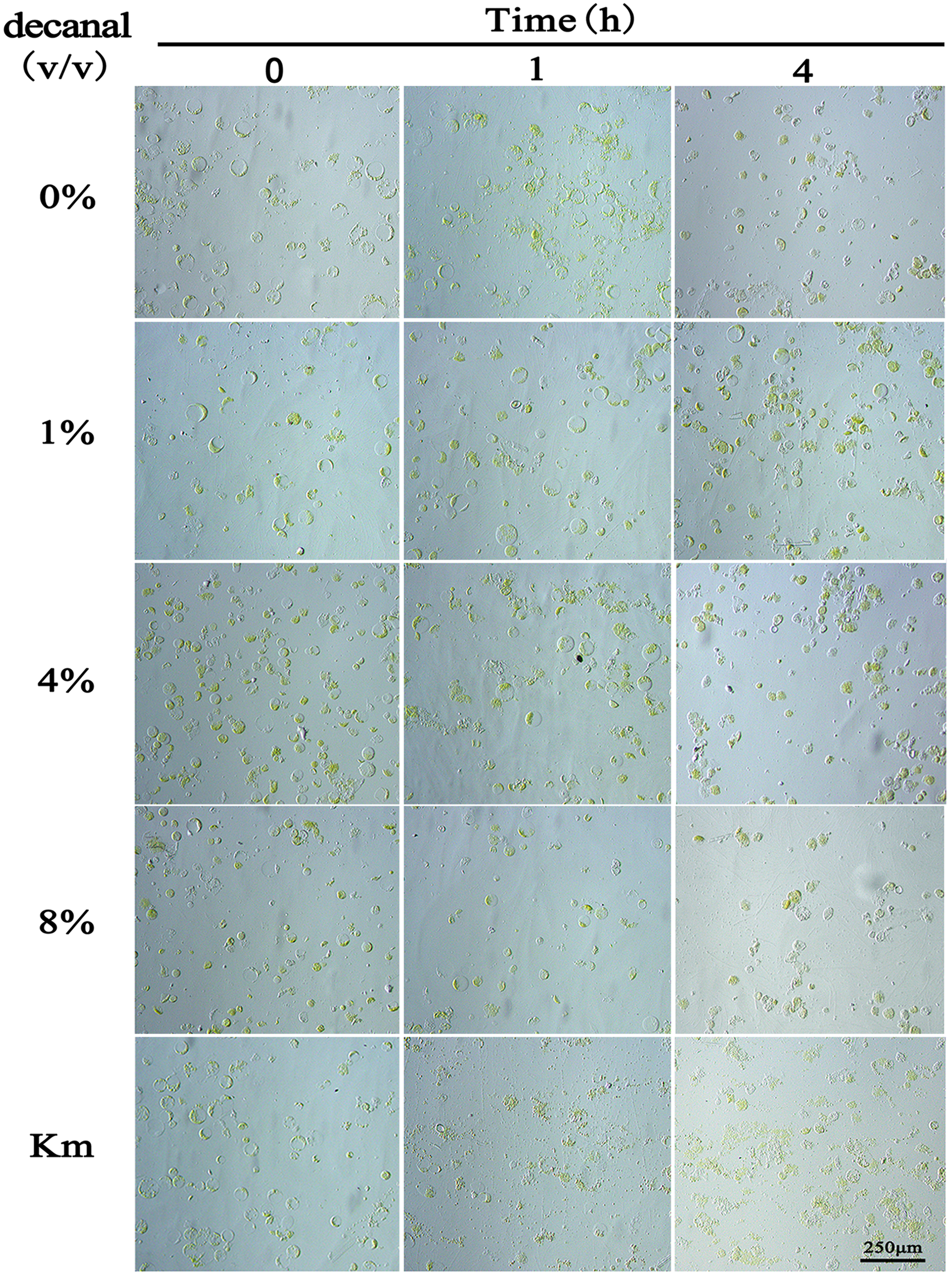

Supplement: Figure S3 — The tolerance of Arabidopsis protoplasts to decanal treatment. Protoplasts treated with different concentrations of decanal were photographed under optical microscope at time 0, 1 and 4 hours, respectively. Row 5 shows the protoplast cells tested with 200 µg/ml kanamycin as a control. Kanamycin but not decanal treatment leads to quick lysis of protoplast cells. (TIF) [file pone.0107885.s003.tif]
