# Supplementary material for: Engineering an Enhanced, Thermostable, Monomeric Bacterial Luciferase Gene As a Reporter in Plant Protoplasts
Source: PLoS One. 2014 Oct 1;9(10):e107885. doi: 10.1371/journal.pone.0107885 (PMC4182741; doi:10.1371/journal.pone.0107885)
Supplement: Table S1 — Bacterial strains and plasmids used in this study. (DOCX) [file pone.0107885.s004.docx]

**Table S1.**

| **Strains or plasmids** | **Relevant genotype description** | **References** |
| --- | --- | --- |
| **Strains** |  |  |
| *Y. pseudotuberculosis* YPIII | Wild type *Y. pseudotuberculosis* pIB1, Nal^r^ | [[1](#_ENREF_1)] |
| *E. coli* DH5α | Host for cloning or expression vector pBluescript and pKT100 | TaKaRa |
| BL21(DE3) | Host for expression vector pET28a | Novagen |
| *C. glutamicum* RES167 | Restriction-deficient mutant of ATCC13032, Δ(cglIM-cglIR-cglIIR) | [[2](#_ENREF_2)] |
| Yeast(PJ69-4A) | *MATa GALI-HIS3 GAL2-ADE8 GAL7-lacZ leu2 ura3 his3 gal4 gal80* | [[3](#_ENREF_3)] |
| **Plasmids** |  |  |
| pDM4-luxCDABE | *luxCDABE* cassette in plasmid pDM4 | [[4](#_ENREF_4)] |
| pBluescript II SK^+^ | Cloning vector, Amp^r^ | Stratagene |
| pXMJ19 | Shuttle vector (Ptac lacIq pBL1 oriVC. glutamicum pK18 oriV*E. coli*) | [[5](#_ENREF_5)] |
| p425GPD | Amp^r^, leu+, GPD promoter | [[6](#_ENREF_6)] |
| pET28a | Km^r^ | Novagen |
| pBS-T6SS4 | *T6SS4* promoter in pBluescript II SK^+^ | This study |
| pBS-pLac | *lac* promoter in pBluescript II SK^+^ | This study |
| pBS-pLac::luxAB | *luxAB* under the control of *lac* promoter in pBluescript II SK^+^ | This study |
| pBS-pLac::eluxAB | e*luxAB* under the control of *lac* promoter in pBluescript II SK^+^ | This study |
| pBS-pT6SS::luxAB | *luxAB* under the control of *T6SS4* promoter in pBluescript II SK^+^ | This study |
| pBS-pT6SS::luxA+B | *luxA+B* under the control of *T6SS4* promoter in pBluescript II SK^+^ | This study |
| pBS-pT6SS::eluxAB | e*LuxAB* under the control of *T6SS4* promoter in pBluescript II SK^+^ | This study |
| pET28a-luxAB | *luxAB* under the control of *T7* promoter in pET28a | This study |
| pET28a-luxA+B | *luxA+B* under the control of *T7* promoter in pET28a | This study |
| pXMJ19-luxAB | *luxAB* under the control of *tac* promoter in pXMJ19 | This study |
| pXMJ19-eluxAB | e*luxAB* under the control of *tac* promoter in pXMJ19 | This study |
| p425GPD-luxAB | *luxAB* under the control of *GPD* gene promoter in p425GPD | This study |
| p425GPD-eluxAB | *luxAB* under the control of *GPD* gene promoter in p425GPD | This study |
| p425GPD-opt-eluxAB | opt-e*luxAB* under the control of *GPD* gene promoter in p425GPD | This study |
| pGL3-dual luciferase | Amp^r^  2×35S promoter, as a carrier of the plant report of lux vectors(a1, a2, a3, a4, a5) | [[7](#_ENREF_7)] |
| pCAMBIA3301 | Contain the fragement of 35S | [[8](#_ENREF_8)] |
| pGL3-eluxA+B | e*luxA+B* under the control of 2×35S promoter in pGL3 | This study |
| pGL3-eluxAB | e*luxAB* under the control of 2×35S promoter in pGL3 | This study |
| pGL3-opt-eluxA+B | opt-e*luxA+B* under the control of 2×35S e promoter in pGL3 | This study |
| pGL3-opt-eluxAB | opt-e*luxAB* under the control of 2×35S promoter in pGL3 | This study |
| pGL3-opt-35S-eluxAB | opt-e*luxAB* under the control of 35S promoter in pGL3 | This study |
| pGL3-opt-non-eluxAB | opt-e*luxAB* without promoter in pGL3 | This study |

*Nal^r^, Km^r^ and Amp^r^ represent resistance to naladixic acid, kanamycin and ampicillin at 15, 50 and 100 μg/ml, respectively.

**References**

1. Rosqvist R, Skurnik M and Wolf-Watz H (1988) Increased virulence of *Yersinia pseudotuberculosis* by two independent mutations. Nature. 334: 522-524.
2. Tauch A, Kirchner O, Löffler B, Götker S, Pühler A, et al. (2002) Efficient electrotransformation of *Corynebacterium diphtheriae* with a mini-replicon derived from the *Corynebacterium glutamicum* plasmid pGA1. Curr. Microbiol. 45: 362-367.
3. James P, Halladay J and Craig EA (1996) Genomic libraries and a host strain designed for highly efficient two-hybrid selection in yeast. Genetics. 144: 1425-1436.
4. Milton DL, Hardman A, Camara M, Chhabra SR, Bycroft BW, et al. (1997) Quorum sensing in *Vibrio anguillarum*: characterization of the vanI/vanR locus and identification of the autoinducer N-(3-oxodecanoyl)-L-homoserine lactone. J. Bacteriol. 179: 3004-3012.
5. Jakoby M, Ngouoto-Nkili C-E and Burkovski A (1999) Construction and application of new *Corynebacterium glutamicum* vectors. Biotechnology Techniques. 13: 437-441.
6. Mumberg D, Müller R and Funk M (1995) Yeast vectors for the controlled expression of heterologous proteins in different genetic backgrounds. Gene. 156: 119-122.
7. Gu L, Han Z, Zhang L, Downie B and Zhao T (2013) Functional analysis of the 5' regulatory region of the maize GALACTINOL SYNTHASE2 gene. Plant Sci. 213: 38-45.
8. De Block M, Botterman J, Vandewiele M, Dockx J, Thoen C, et al. (1987) Engineering herbicide resistance in plants by expression of a detoxifying enzyme. EMBO J. 6: 2513.
