# Supplementary material for: Engineering an Enhanced, Thermostable, Monomeric Bacterial Luciferase Gene As a Reporter in Plant Protoplasts
Source: PLoS One. 2014 Oct 1;9(10):e107885. doi: 10.1371/journal.pone.0107885 (PMC4182741; doi:10.1371/journal.pone.0107885)
Supplement: Table S2 — Primers used in this study. (DOC) [file pone.0107885.s005.doc]

**Table S2.**

| **Primers** | **Sequence (5' to 3')** |
| --- | --- |
| LuxA-XbaI-F | GCTCTAGAATGAAATTTGGAAACTTTTTGC |
| LuxA-L_15_-R | GCTACCTCCGCCACCACTTCCACCGCCTCCAGAACCTCCTCCACCATATAATAGCGAACGTTGTTTTTC |
| LuxB-L_15_-F | GGTGGAGGAGGTTCTGGAGGCGGTGGAAGTGGTGGCGGAGGTAGCATGAAATTTGGATTGTTCTTCC |
| LuxB-BglII-R | GAAGATCTTTAAGCGGCCGCCATATGGGATCCGGTATATTCCATGTGGTACTTC |
| LuxB-BamHI-R | GACGGATCCTTAGGTATATTCCATGTGGTACTTC |
| LuxA-BamHI-F | CGCGGATCCATGAAATTTGGAAACTTTTTGC |
| LuxB-SalI-R | ACGCGTCGACTTAGGTATATTCCATGTGGTACTTC |
| pLac-SacI-F | CGGGAGCTCGCGCAACGCAATTAATGTG |
| pLac-XbaI-R | CTAGTCTAGAAGCTGTTTCCTGTGTGAAATTG |
| pT6SS4-SacI-F | CGGGAGCTCATATCCCCCTTTGACATTTC |
| pT6SS4-XbaI-R | GCTCTAGAAACGCCGAATAATGCTTGAG |
| nos-F | TACCACATGGAATATACCTAATGCGGCCGCTTCGAGCAGAC |
| nos-SalI-R | ACGCGTCGACTTATCGATTTTACCAC |
| eluxA-NheI-F | CTAGCTAGCATGAAATTTGGAAACTTTTTGC |
| eluxA-XbaI-R | CTAGTCTAGACTAATATAATAGCGAACGTTGTTTTTC |
| eluxB-NcoI-F | CATGCCATGGAATTTGGATTGTTCTTCCTTAAC |
| eluxB-XbaI-R | CTAGTCTAGATTAGGTATATTCCATGTGGTACTTC |
| eluxB-R | TTAGGTATATTCCATGTGGTACTTC |
| opt-eluxA-NheI-F | CTAGCTAGCACATTTGCTTCTGACAC |
| opt-eluxA-XbaI-R | CTAGTCTAGAACTAGTACAAAAGTGACCTTTG |
| opt-eluxB-NcoI-F | CATGCCATGGGTAAGTTTGGACTCTTTTTCT |
| opt-luxB-XbaI-R | CTAGTCTAGAATTATGTGTATTCCATGTGG |
| opt-eluxB-R | TTATGTGTATTCCATGTGG |
| 35S-KpnI-F | CGGGGTACCCATGGAGTCAAAGATTCAAATAG |
| 35S-NheI-R | CTAGCTAGCAGTCCCCCGTGTTCTCTC |

*Underlined sequences indicate the L_15_ linker encoding regions.
