# Supplementary material for: Engineering an Enhanced, Thermostable, Monomeric Bacterial Luciferase Gene As a Reporter in Plant Protoplasts
Source: PLoS One. 2014 Oct 1;9(10):e107885. doi: 10.1371/journal.pone.0107885 (PMC4182741; doi:10.1371/journal.pone.0107885)
Supplement: Table S3 — Comparison of luminescence activities between bacterial, Renilla and firefly luciferases in plant protoplasts. Luminescence activities were measured in Arabidopsis and maize protoplast cells transformed with Rluc and Fluc reporter vector (pGL3-dual luciferase) or with opt-eluxAB reporter vector (pGL3-opt-eluxAB). The number of Arabidopsis protoplasts used was about 5×10^6^ and of the maize protoplasts was about 3×10^5^ in 100 µl volume culture. (DOCX) [file pone.0107885.s006.docx]

**Table S3**

| Reporter vectors | RLU（relative luminescence units） | |
| --- | --- | --- |
|  | *Arabidopsis* | Maize |
| Fluc | 2.6 (±0.2) × 10^6^ | 1.0 (±0.2) × 10^6^ |
| Rluc | 6.0 (±2.0) × 10^4^ | 2.0 (±0.3) × 10^4^ |
| Opt-eluxAB | 1.8 (±0.2) × 10^4^ | 1.5 (±0.1) × 10^3^ |
